# Supplementary material for: Identification of photoperiod-induced specific miRNAs in the adrenal glands of Sunite sheep (Ovis aries)
Source: Front Vet Sci. 2022 Jul 22;9:888207. doi: 10.3389/fvets.2022.888207 (PMC9354845; doi:10.3389/fvets.2022.888207)
Supplement: Supplementary file 1 [file Data_Sheet_1.ZIP › Supplementary file/miRDeep2 file.zip!/miRDeep2 file/Know_Structure/SP42A3/oar-mir-431.pdf]

miRBase precursor : oar-mir-431
Total read count : 111
oar-miR-431 read count : 15
remaining reads : 96

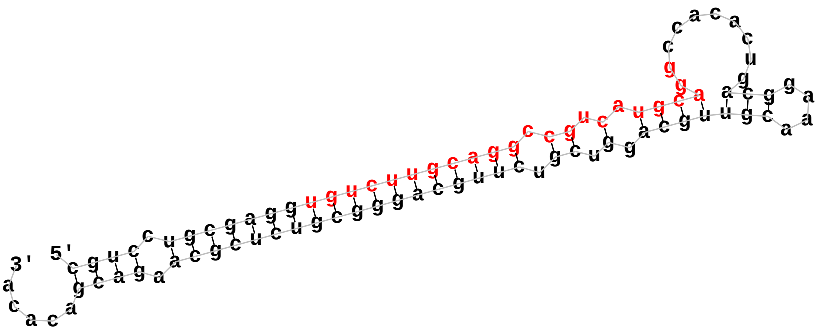

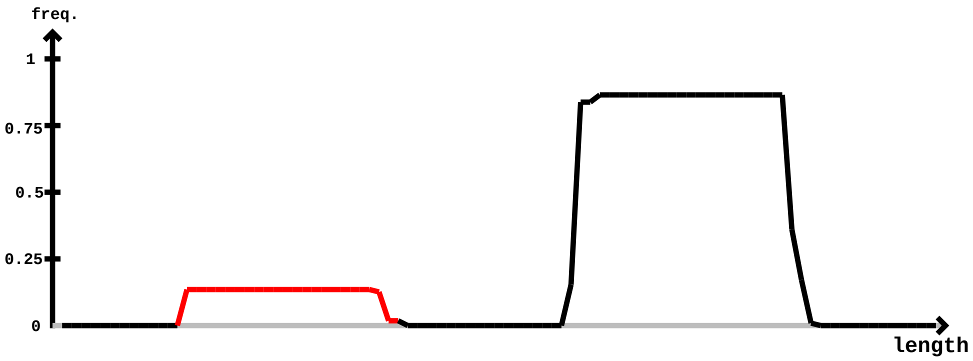

oar-miR-431

```
5'- cguccugcgaggugucuugcaggccgucaugcaggccacacugacggaaacguugcaggucgucuugcagggcgucucgcaagacgacaca   -3'    exp
    ((((.((((((((((((((((((.((.(.((((..........(((....))))))).).)).)))))))))))))))))).)))).....    reads      mm         sample
    ...........ugucuugcaggccgucaugc............................................................    1          0          seq
    ...........ugucuugcaggccgucaugca...........................................................    12         0          seq
    ...........ugucuugcaggccgucaugcagg.........................................................    2          0          seq
    ....................................................uugcaggucgucuugcagggcgu................    13         0          seq
    ....................................................uugcaggucgucuugcagggcguc...............    1          0          seq
    ....................................................uugcaggucgucuugcagggcgucu..............    3          0          seq
    .....................................................ugcaggucgucuugcagggcgu................    41         0          seq
    .....................................................ugcaggucgucuugcagggcguc...............    19         0          seq
    .....................................................ugcaggucgucuugcagggcgucu..............    15         0          seq
    .....................................................ugcaggucgucuugcagggcgucuc.............    1          0          seq
    .......................................................caggucgucuugcagggcgu................    2          0          seq
    .......................................................caggucgucuugcagggcguc...............    1          0          seq
```
